# VIEWS OF OLDER ADULTS ON SUPPORT OPTIONS TO RELIEVE THEIR WORKING INFORMAL CAREGIVERS: A STORY COMPLETION STUDY

**DOI:** 10.1093/geroni/igac059.2943

**Published:** 2022-12-20

**Authors:** Eline Vos, Simone de Bruin, Suzanne Pees, Allard van der Beek, Karin Proper

**Affiliations:** National Institute for Public Health and the Environment, Bilthoven, Utrecht, Netherlands; Windesheim University of Applied Sciences, Zwolle, Overijssel, Netherlands; National Institute for Public Health and the Environment, Bilthoven, Utrecht, Netherlands; Amsterdam Public Health research institute, Amsterdam UMC, Amsterdam, Noord-Holland, Netherlands; National Institute for Public Health and the Environment, Bilthoven, Utrecht, Netherlands

## Abstract

Older care recipients living at home increasingly rely on support from informal caregivers, who often combine their caregiving tasks with a paid job. Because of this combination of tasks, working caregivers may become overloaded. Different caregiver support options are available, that also affect older care recipients’ lives, such as respite care, technology, or home care. The aim of this qualitative study was to obtain insight into the perceptions, concerns and preferences of older informal care recipients about the use of support to relieve their working informal caregivers. We performed a story completion writing task among 23 informal care recipients aged 65 years or older, using hypothetical situations to let participants reflect on different caregiver relief support options. The task was followed-up by a story-mediated interview. We used thematic analysis to inductively analyze story completions and interviews. We found that older adults were often willing to use support to relieve their caregivers, to ensure their wellbeing. However, they were also concerned about how the use of such support may conflict with their interests, preferences and values (e.g. respect for their autonomy, privacy, having a trusting relationship with caretakers, paying attention to human aspects in care). Older adults were most hesitant to use adult day care facilities and technological support options. While these areas of tension cannot always be completely resolved, it is important to jointly identify and discuss these, and work towards solutions to balance the respective interests, values and needs of older adults and caregivers.

